# Specific Targeting of Notch Ligand-Receptor Interactions to Modulate Immune Responses: A Review of Clinical and Preclinical Findings

**DOI:** 10.3389/fimmu.2020.01958

**Published:** 2020-08-14

**Authors:** Mounika U. L. Goruganthu, Anil Shanker, Mikhail M. Dikov, David P. Carbone

**Affiliations:** ^1^Department of Internal Medicine, James Comprehensive Cancer Center, The Ohio State University Wexner Medical Center, Columbus, OH, United States; ^2^Department of Biochemistry, Cancer Biology, Neuroscience and Pharmacology, Meharry Medical College School of Medicine, Nashville, TN, United States; ^3^Vanderbilt-Ingram Cancer Center, Nashville, TN, United States

**Keywords:** Notch signaling, Notch therapeutics, engineered Notch ligands, cancer immunotherapy, immunosurveillance, T cells, antigen presenting cells, tumor escape

## Abstract

Understanding and targeting Notch signaling effectively has long been valued in the field of cancer and other immune disorders. Here, we discuss key discoveries at the intersection of Notch signaling, cancer and immunology. While there is a plethora of Notch targeting agents tested *in vitro*, *in vivo* and in clinic, undesirable off-target effects and therapy-related toxicities have been significant obstacles. We make a case for the clinical application of ligand-derived and affinity modifying compounds as novel therapeutic agents and discuss major research findings with an emphasis on Notch ligand-specific modulation of immune responses.

## Introduction

Notch signaling plays a variety of physiological roles including, but not limited to, cell proliferation, cell fate decisions, cellular differentiation and angiogenesis (1). The role and importance of Notch signaling in hematopoietic compartment now stands undisputed. Despite the improved clinical response compared to standard chemotherapy, the efficacy of immune checkpoint inhibitors (ICI) across a variety of solid tumors is limited to a fraction of patients (2, 3). It is therefore essential to develop therapeutic agents that show promise as single agent immunomodulators or can be used in combination with ICIs to elicit antitumor immune responses.

Developing and utilizing agents that could support the induction of antitumor T cell functions while also precluding effector immune cells from immunosuppression offers great promise. Findings from murine models of solid tumors, allergic responses and autoimmune disorders indicate great potential for the clinical application of Notch ligands and their derivatives as immunomodulatory agents for the management of malignant cancers (4, 5). Engineered Notch ligand-derived moieties could be used to induce desired immune responses and boost antitumor immunity (6, 7).

Activating mutations in Notch1 have been described in lung, breast, colorectal and pancreatic cancers to name a few. On the other hand, loss of function mutations in Notch in hepatocellular carcinomas and melanomas have established its role as a tumor suppressor (8). Notch can play a highly contextual role in tumoral, stromal and immune compartments, which adds to the signaling complexity and warrants the need to pursue its therapeutic targeting with great prudence.

In the following sections, we report findings that revealed the varied effects of Notch signaling in immune compartments driving T cell development, activation, differentiation, and regulation of effector immune responses. Non-canonical Notch signaling and its crosstalk with other signaling pathways, impact of Notch post-translational modifications on T cell differentiation, consensus and controversies and open questions in the field are discussed. We highlight how knowledge obtained by structural studies and studying the mechanisms of various steps involved in Notch activation and signal transduction offer therapeutic opportunities that enable its targeting with high specificity.

## Brief Overview of Notch Signaling

Canonical Notch signaling is unique in being driven by juxtracrine cell membrane bound receptor-ligand interactions (9). The mammalian Notch system is comprised of four Type I transmembrane receptors (Notch1-4) and two classes of ligands – Delta-like (DLL 1,3,4) and Jagged (JAG 1,2). Upon ligand binding, a mechanical force triggers sequential proteolytic cleavages in the intracellular portions of the receptor, ultimately releasing the Notch intracellular domain (NICD) into the cytoplasm. NICD then migrates into the nucleus where it acts along with a host of other transcriptional coactivators, including RBP-Jκ and MAML1-3 (10). To ensure tight regulation of Notch signaling, C-terminal PEST domain provides a proteolytic target for degradation of active Notch (11–13). Recent developments have indicated that Notch can also exert its functions non-canonically by interacting with members of other signaling pathways such as Wnt/β-catenin, NF-κB, TGFβ and many others (14–19).

## Role of Notch Signaling in Helper and Effector T Cells

Select Delta-like ligands have been shown to induce differentiation of hematopoietic stem cells (HSCs) into T cells. These findings were obtained using OP9 bone marrow stromal cells expressing DLL1 or DLL4 and similar effects were also observed using purified plate-bound ligands (20, 21). T cell differentiation of HSCs is dependent on both ligand identity and level of expression where low-level expression of the Delta-like ligands attenuates but does not eliminate the myeloid potential of HSCs. Such fine tuning of dose responses is a recurring theme in Notch signaling, faithful artificial recapitulation of which has eluded us so far.

Antigen presenting cells (APCs) expressing Delta-like ligands activate and polarize naïve CD4^+^ T cells to a Th1 phenotype, while JAG1/2 expressing APCs lead to Treg/Th2/Th17 polarization (22–24). Convincing results in this direction showed that the intracellular domain of Notch1 is directly involved in interactions with and expression of Th1 master transcriptional factor T-bet and production of cytokine IFNγ in CD4 T cells. Notch signaling promoted the development of CD8^+^ terminal effector T cells and suppressed memory-precursor fate in effector-memory T cell (T_EM_) subsets (25). Activation of the Notch pathway in T_EM_ cells also suppressed memory-precursor fate. Transcription factors such as eomesodermin (EOMES) and T-bet were found to be directly regulated by Notch, further supporting the importance of Notch signaling in driving effector T cell responses (26–28).

The tumor microenvironment (TME) also plays a major role in influencing T cell responses. Notch1 and Notch2 were found to be downregulated in tumor infiltrating T cells but not in splenic T cells of tumor-bearing mice (29). This attenuation of infiltrating T cell responses was driven by Jag1/2 expressed by immunosuppressive myeloid-derived suppressor cells (MDSCs) which could be overcome by ectopic expression of Notch1 intracellular domain (N1ICD) in antigen specific T cells, indicating that the TME is programmed with mechanisms to suppress Notch signaling and evade T cell-mediated tumor cell death. This reveals another interesting aspect of Notch: the spatio-temporal regulation of Notch ligand and receptor expression.

**FIGURE 1:**
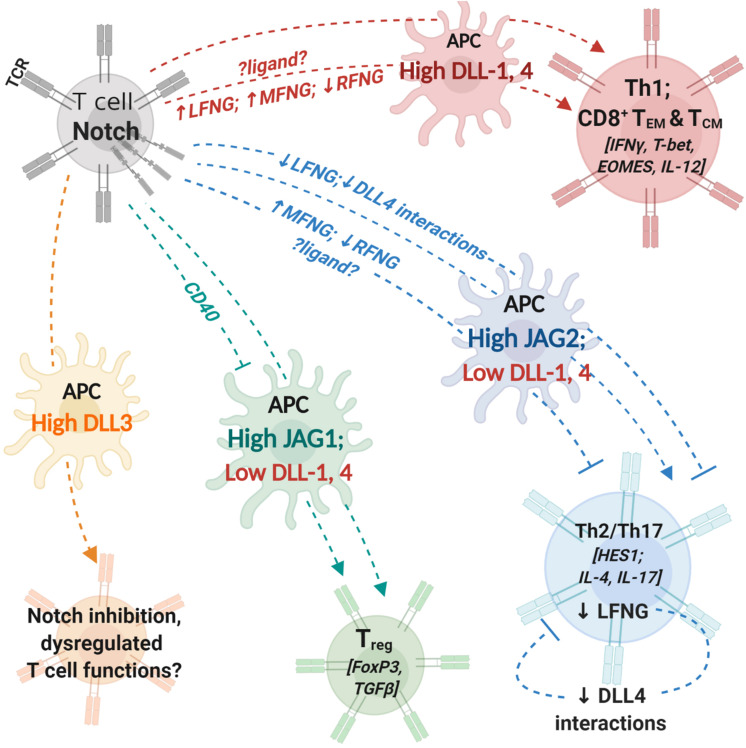
Notch interactions between antigen-presenting cells and T cells influence helper and effector T cell activity. T cells express T cell receptor (TCR) complex and Notch receptors. Antigen-presenting cells (APCs) express costimulatory molecules and Notch ligands. During T cell activation, the identity of Notch ligand present on the cell surface of APCs can influence T cell polarization and differentiation. Changes in expression levels of fringe glycosyl transferases can influence the process by modifying Notch receptor affinity to different ligands. Notch signaling in T cells regulates expression of transcription factors and cytokines (indicated within []) involved in helper and cytotoxic T cell functions. APCs with high expression levels of DLL1 or DLL4 can polarize CD4^+^ T cells into aTh1 phenotype and drive CD8^+^ T cell differentiation into memory cells. Increase (↑) in LFNG and MFNG expression and downregulation/loss (↓) of RFNG expression can enhance Th1 differentiation; identity of ligands involved in fringe-mediated Th1 differentiation are yet to be investigated (represented by ?ligand?). APCs with high JAG2 and low DLL1,4 expression drive helper T cell differentiation into Th2 or Th17 phenotypes. Expression of MFNG and downregulation of RFNG can block Th2 differentiation. Loss of LFNG in uncommitted T cells as well as Th2 polarized cells inhibits Notch interactions with DLL4 and attenuates Th2 responses. APCs with high JAG1 expression can induce T cell polarization into regulatory T cells (T_reg_). CD40 blockade together with JAG1 expression on APCs enhances immunosuppressive functions of T_reg_ cells. APC, antigen presenting cell; DLL, Delta-like ligand; JAG, Jagged ligand; LFNG, lunatic fringe; MFNG, manic fringe; MHC, major-histocompatibility complex; TCR,; Th1, T helper type 1, Th2: T helper type 2; Th17, T helper type 17; T_reg_, T regulatory cell; T_EM_, effector-memory T cell; T_CM_, central-memory T cell; RFNG, radical fringe.

While several studies demonstrated the involvement of Notch signaling in driving effector and helper T cell responses (summarized in Figure 1), the precise regulatory mechanisms behind cell surface expression of Notch ligands and receptors are only partly known. TCR stimulation has been shown to induce expression of Notch1, Notch2 and Hes1 (Notch target) in T cells (30) but T cell activation using CD28 beads alone or low-dose CD3 and CD28 stimulation induces expression of Notch ligands on T cells (31). Notch ligands were not expressed by T cells during *in vitro* activation with mature bone-marrow derived dendritic cells. The induced ligands also co-localized with Notch receptors on the surface indicating cis-inhibition. Notch ligand expression was abrogated in the presence of NF-κB inhibitor, demonstrating the combined role of Notch and NF-κB pathways in driving T cell functions downstream of TCR stimulus. The observations suggest additional regulatory mechanisms, possibly to prevent erroneous T cell activity in the absence of both TCR and co-stimulatory CD28 signals.

Notch extracellular domain (NECD) binding to cognate ligands is influenced by a variety of post-translational modifications, prominent among them being O-linked glycosylation by Fringe glycosyl transferases (32, 33). The three mammalian fringe proteins, Lunatic (Lfng), Manic (Mfng) and Radical (Rfng) extend *O*-Fucose moieties with GlcNAc at conserved serine or tyrosine residues in EGF repeats of NECD (34, 35). Glycosylation of Notch by Lfng and Mfng enhances interactions with Delta-like ligands while suppressing interactions with Jagged ligands. On the other hand, Notch glycosylation by Rfng enhances receptor interactions with both classes of ligands.

Tumor-mediated decrease in Lfng and Mfng expression levels have been shown to promote metastasis and poor survival (36, 37). Lfng interacts cooperatively with p53 to suppress tumors and Mfng suppresses tumorigenic activity of JAG1 and Notch3 (38–40). Lfng and Mfng thus appear to have a tumor-suppressive role in solid tumors and restoring their expression levels can be pursued as a therapeutic strategy to achieve tumor regression.

Fringe-mediated changes in Notch ligand-receptor interactions lead to dysregulations in thymic and ectopic T cell development resulting in altered T/B cell population ratios (41–44). Tumor burden and tumor-derived immunosuppressive cytokines also cause abnormalities in intrathymic T cell differentiation and development (45–47). Notch glycosylation by fringes influence the differentiation of mature T cell populations as well. It was found that Lfng and Mfng were downregulated and Rfng was upregulated in naïve CD4^+^ T cells in asthmatic rats (48). This was associated with more active Notch signaling in asthmatic naïve CD4^+^ T cells compared to control naïve T helper cells. Restoring Lfng and Mfng expression and silencing Rfng enhanced the number of Th1 cells while lowering Th2 cell differentiation. Lfng overexpression in naïve CD4^+^ T cells was able to drive Th1 and Th2 differentiation in a Notch-independent and dependent manner, respectively. These findings indicate that modulating Fringe expression levels can be potential therapeutic strategies for the management of allergic diseases. Moreover, the observation that fringe expression levels vary even in naïve CD4^+^ T cells under asthmatic conditions provide a basis for the hypothesis that there might be molecular factors that can alter T cell programs, which need to be elucidated. While Gu et al., were able to demonstrate the role of fringe glycosylation in influencing T helper cell differentiation, the source and identity of Notch ligands involved in this process were not identified.

Using a mouse airway allergic disease model, another study found that transcription of Lfng was driven by STAT5 in Th2 helper cells (49). Th2-mediated airway hyper-reactivity, mucus production and IL4 production was driven by DLL4-mediated Notch activation. Specifically, deletion of Lfng but not Mfng or Rfng in Th2 and CD4^+^ T cells resulted in reduced Th2 responses and inflammation. While STAT5 and GATA3 were previously known to drive Th2 differentiation independent of Notch signals (50, 51), the regulation of Lfng expression by STAT5 in Th2 subsets is a novel and interesting finding. It is likely that other inflammatory factors that can influence STAT5 signaling can potentially alter fringe expression levels. Notch activity in T cells thus can be profoundly influenced by complex intracellular networks of cytokines and signaling pathways involved in fine-tuning immune responses (52, 53).

## Contrasting Observations and an Argument for Non-Canonical Notch Signaling in T Cells

Differentiation of complete T cell effector program has been observed to be dictated by the identity of Notch ligand expressed on APCs, which in turn is dictated by the type of antigenic stimulus encountered (22, 23). This is in stark contrast to observations from *in vitro* T cell differentiation by polarizing cytokines even in the absence of Notch ligands (54). In some *in vitro* experiments, Notch activity was shown to confer a proliferative effect in T cells but could not drive Th1/Th2 differentiation in the absence of polarizing cytokines (55). While some studies have demonstrated that DLL1/4 ligands can promote a Th1 polarization, others have argued that the Th1 phenotype is not acquired as a consequence of Notch signaling but by suppression of the alternative Th2/17 fate (56, 57). The disease model used, type of antigenic responses, stimuli involved in DC maturation and the relative expression levels of different Notch ligands are all factors that could potentially influence T cell polarization by APCs. Most studies, however, have produced convincing data in favor of Notch1-ICD binding directly to promoters of genes and transcription factors driving Th1 and cytotoxic responses. Non-canonical Notch signaling and crosstalk with NF-κB pathway is also observed in activated T cells (58). γ-secretase inhibitors reduced IFNγ production in *in vitro* activated CD8^+^ T cells but not in CD4^+^ cells, which can indicate that helper and cytotoxic T cells respond differently to Notch stimuli at least *in vitro*. It is likely that DC-borne ligands could orchestrate T cell survival and proliferation within an existing cytokine milieu instead of having an instructive role in naïve T cell differentiation (59–61).

These observations prompt a question: do Notch ligands play a deterministic/instructive role or do they simply enhance pre-existing T cell programs in an unbiased manner? It could be possible that Notch serves as a costimulatory signal that can set in motion any of the numerous downstream signaling pathways (62, 63). It might also be possible that Notch signaling might have different effects before, during and after T cell activation and differentiation. Majority of the studies on the role of Notch in immune cell functions have looked at Hes/Hey/Deltex family members, which are themselves transcriptional factors effecting expression of several genes. T cell functions might be ultimately dictated by a combinatorial framework in which terminal effector molecules are further regulated by Notch targets.

## Unanswered Questions

Notch signaling does not always appear to operate as a simple ON/OFF switch. It has been shown to be regulated by a complex system of fine-tuning and crosstalk of input signals including relative expression levels of ligands and receptors, numerous post-translational modifications and a combination of cis- and trans- interactions (64–67). While attempts are being made to target Notch in various disease settings, a large number of therapies developed so far have led to undesirable side-effects and toxicities (7). To address these shortcomings, it is important to study the mechanistic and physical aspects of ligand-receptor interactions (68) and role of post-translational modifications such as ligand glycosylation and ubiquitination (32, 33, 35). It is also necessary to understand how the physiological consequences of ectopic Notch expression are similar to and differ from ligand-specific receptor activation and how different sources of ligands can influence differences in immunological outcomes. Redundancies in receptor and ligand paralogs also need to be resolved.

## Therapeutic Strategies to Target Notch Signaling

**FIGURE 2:**
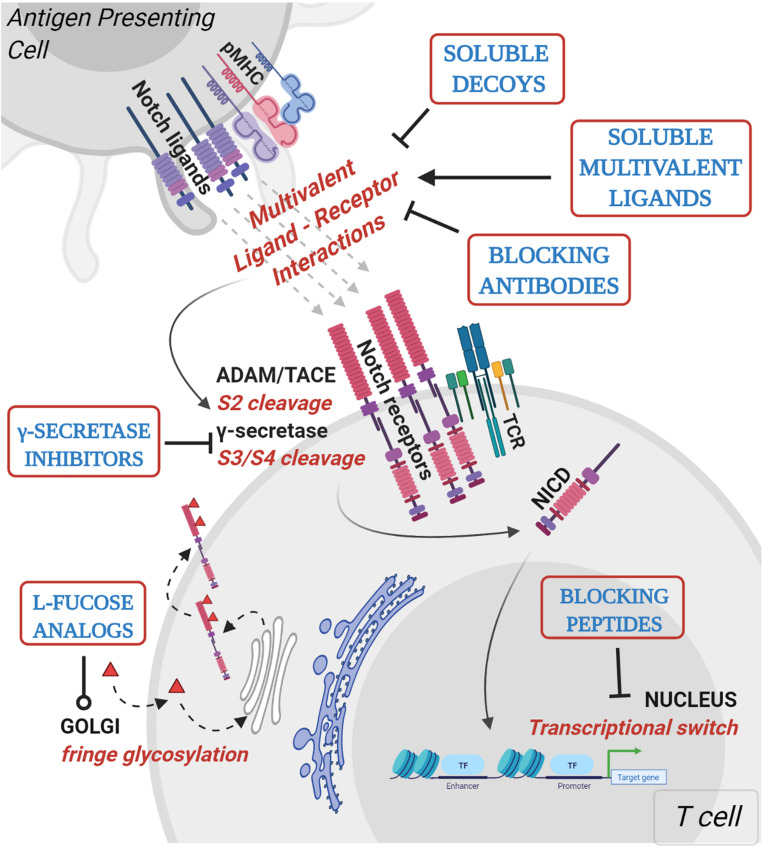
Mechanistic basis for therapeutic targeting of ligand-specific Notch signaling. Agents targeting Notch signaling can be grouped by the step or process in the Notch signaling pathway that is being affected. Soluble decoys comprise of extracellular portions of Notch ligands or receptors that can competitively inhibit multivalent receptor-ligand interactions. Soluble multivalent ligands comprise of clustered ligands that provide and/or augment ligand-specific Notch activation. Blocking antibodies block receptor interactions with ligands and are paralog-specific antagonists with high selectivity. γ-secretase inhibitors prevent NICD release by inhibiting S3 cleavage of Notch receptors at the juxtamembrane domain. L-fucose analogs (solid red triangles) are taken up by cells from media and incorporated into receptor extracellular domains. Fucose analogs on Notch receptors alter ligand-binding affinities and can be used to block selective ligand interactions. Blocking peptides target protein-protein interface in the nuclear Notch transcriptional complex and prevent transcription of Notch target genes. *In vitro*, pre-clinical and clinical studies demonstrating Notch-modulatory activities and anti-tumor efficacy of various classes of Notch therapeutics are presented in Table 1.

Knowledge-based approaches on the activation mechanisms of Notch have led to the development of several Notch inhibitory agents. These include selective ligand/receptor-specific decoys, agents that block receptor cleavage, molecules that inhibit formation of Notch-CSL activator complex, antibodies, and post translational modifications influencing ligand-receptor interactions (Table 1 and Figure 2). In addition to being used as single agents in various clinical and preclinical studies, Notch inhibitors are also being studied in combination with current chemotherapeutic drugs. Despite being uncharacterized for the active component, some natural compounds show promising anti-proliferative effects on cancer cell lines and have traditionally been used as part of dietary modifications as chemo-preventative measures (69–71). Inhibition of the γ-secretase complex is the most widely employed method of blocking Notch signaling but has been fraught with toxicities (72, 73). There is a need to focus on Notch activators in the management of cancers like lung squamous cell carcinoma, where Notch acts as a tumor suppressor. The development of Notch modulators should be guided at every stage by the biological and physiological effects of the compounds being tested. Mechanism-based combinatorial regimens, biomarkers of response and contextual frameworks need to be developed and evaluated on a case by case basis.

**TABLE 1:** Strategies to target Notch signaling.

Class	Agent(s)	Target	Mechanism	Cancer type; *in vivo/in vitro* model	Treatment-related toxicities	References
GSI	PF03084014, MK0752	γ-secretase complex	Juxtamembrane cleavage and NICD dissociation	T-ALL*, breast*, lung adenocarcinomas*, thyroid*, prostate*, CNS malignancies*	Gastrointestinal toxicities, diarrhea, nausea, rash, fatigue	(140), NCT00645333, NCT01098344
	A5226A	Nicastrin	Inhibition of γ-secretase activity	Lymphoblastic leukemia^t^, NSCLC^t^	na	(141)
Blocking peptides	SAHM1	MAML1	Direct binding to pre-assembled Notch1–CSL/RBP-Jκ complexes and competitive inhibition of the MAML1 co-activator binding	T-ALL^t^, murine asthma model	na	(142, 143)
Blocking antibodies	OMP-59R5, anti-NRR1, anti-NRR2	Notch1, Notch2, Notch3	Blocking receptor mediated signaling	Stage IV NSCLC*, extensive stage small-cell lung cancer*	Atrial fibrillation, diarrhea	PINNACLE (NCT01859741), (144, 145)
	OMP-21M18, REGN421	hDLL4	Humanized antibody that blocks DLL4 interactions with Notch	Breast^t^, colon^t^, ovarian^t^, pancreatic^t^, NSCLC^t^ & patients with advanced malignancies*	Hypertension, congestive heart failure	NCT01189968, NCT01189929, NCT00871559, (146, 147)
Decoys [soluble ligand or receptor forms]	N1_1–24_	DLL1,4	Pan ligand blocking	Mammary, pancreatic, lung and melanoma tumor models	na	(28)
	N1_1–36_	JAG1,2				
	N1_1–13_	DLL1,4	Specific blocking of Delta-like ligands			
	N1_10–24_	JAG1, 2	Specific blocking of Jagged ligands			
	sJ1, sJ1_N–E3_	JAG1	Endogenous Jagged1	LLC		(6)
L-Fucose analogs	6-alkynyl and 6-alkenyl fucose	Notch ECD fucosylation	Substrate for POFUT-1 incorporated into Notch1 ECD, preventing binding to DLL1,4	T cell differentiation model [OP9 stromal coculture]	na	(98)
Soluble multivalent ligands	cDLL1	Notch1-4	Provides DLL1 stimulus to activate Notch receptors	Lung tumor models, *in vitro* mouse and human T cell cultures	na	(2, 5)

Examples of Notch-targeting agents used in *in vitro*, pre-clinical and clinical studies. *, tested in clinical trial; t, preclinical/*in vitro* data.

While most reagents presented in Table 1 were initially used to alter Notch signaling in stroma and the tumor microenvironment, recent focus has shifted to targeting Notch in tumor-infiltrating and circulating immune compartments. This has been done using agents directly targeting Notch receptors expressed by immune cells or in *in vitro* settings where Notch ligand-based agents are employed to activate, prime, and expand helper and effector T cell populations.

## Notch-Based Reagents for Adoptive T Cell Therapy

As the biology of Notch signaling in driving T cell development began to be better understood, the system was applied to generate antigen-specific T cells *in vitro*. By coculturing with DLL1-expressing bone marrow stromal cells, embryonic and hematopoietic stem cells could be differentiated into immunocompetent T lymphocytes (74). NY-ESO-1–specific and human p53–specific, HLA-A2–restricted human TCR vectors were used to transduce human umbilical cord HSCs, which were then cultured on OP9-GFP or OP9-DL1 cells and expanded (75). The T cells thus generated displayed very little endogenous TCR and had a high expression of antigen-restricted tumor-reactive TCR. They were also less differentiated than *in vitro* expanded lymphocytes that are currently employed in clinic. The differentiated and expanded HSCs in this study expressed the NK cell markers CD56 and CD16 as well as the T cell markers CD3 and CD7 but did not express IFNγ and IL-4 as NK-T cells do. Both NY-ESO1 and p53 TCR-transduced and differentiated cells exhibited antigen-specific lysis of target cells indicating T cell properties. The p53-TCR transduced HSCs, however, lysed both specific and non-specific tumor cells, indicating an NK cell-like behavior. While these cells could be useful candidates for adoptive cell transfer in both HLA-restricted and HLA-independent settings, safety evaluations and detailed characterization of the observed dual T and NK cell behavior is needed. Activated and differentiated effector T cells possess enhanced tumor reactivity *in vitro* but they demonstrate reduced tumor attenuation compared to naïve and early effector cells *in vivo* (76). This was overcome by the generation of stem cell memory T cells [T_SCM_], a class of highly proliferative memory T cells, again using the OP9-DL1 coculture system, to generate ova-specific reactive T cells (77). Of note, the iT_SCM_ cells displayed a loss of PD1 and CTLA4 expression, which contributed in part to enhanced cytolytic activity of the adoptively transferred naïve-like stem cell memory T cells. While Notch1 activity could upregulate PD-1 expression on CD8^+^ T cells activated with artificial APCs expressing both Delta-like and Jagged ligands (78), expression of inhibitory receptors was not seen with the OP9-DL1 coculture system. This indicates the advantage of employing ligand-specific T cell stimulation and expansion for therapeutic applications.

## Notch as a Target of Other Therapeutic Agents and Pathways

Notch signaling has the unique feature of integrating signals from several pathways. This leads to an extensive “hyper-network” situation within a cell as well as at the multicellular level (18, 79). From a therapeutic standpoint, it becomes important to identify key regulatory nodes between different pathways. This will enable the development therapeutic agents with high specificity and prevent cross-pathway side effects. Some studies have identified how Notch in immune cells can be altered by therapeutic and experimental interventions targeting molecules in other signaling pathways.

While T cells can upregulate Notch expression a few hours after TCR stimulation, the exact molecular crosstalk between the two pathways is only partially known (30, 80). PKCθ has been linked to actin regulation as well as Notch induction, leading to the discovery of a spatio-temporal link between T cell stimulation by professional APCs and Notch activity (81). p38 MAPK was shown to induce Jagged1 as well as Notch1 during the maturation of macrophages (82).

Adenosine is an immunosuppressive ATP metabolite that is increased in the extracellular space in response to hypoxia and tissue injury, which can have profound effects on both lymphoid and myeloid cells that express adenosine receptors, predominantly, A2AR by T cells. A2AR agonists have been used in the treatment of inflammatory diseases while A2AR antagonists are being developed as novel cancer immunotherapeutics (83). Notch1 was identified as a target of A2AR-mediated immunosuppression. This is believed to be orchestrated by Cbl-mediated ubiquitination of Notch1 modulated by A2AR via cAMP. CD8^+^ T cells exposed to an A2AR agonist prior to TCR stimulation lowered Notch1 expression, heterodimer cleavage and reduced transcripts of Notch1 target genes *Hes1* and *Myc* (84).

Thus, disparate pathways could potentially converge to drive Notch expression and function. This makes it all the more important to fully understand the fundamental cellular and molecular levels at which Notch signaling is regulated.

## Novel Notch Modulatory Agents

### Engineered Ligand-Specific Therapeutics

An important feature of Notch signaling is the stoichiometry of interactions: activation of Notch receptors requires polyvalent interactions between multiple receptors and ligands. On the other hand, interfering with even a few of the productive multivalent interactions can lead to disruption of Notch signaling. Therefore, soluble monovalent forms of Notch ligands or receptors can potentially act as efficient competitive inhibitors. In contrast, presenting Notch ligands in a multivalent form can provide or enhance ligand-specific stimulus. This mechanistic detail of Notch receptor-ligand interaction can be exploited to design ligand-based reagents that can uniquely stimulate or block Notch signaling.

Studies have demonstrated that endogenous Notch ligand-receptor interactions can be selectively blocked or enhanced to influence signaling in tumoral, stromal and immune compartments (5, 6, 85, 86). Although receptor-ligand interactions can be abrogated using blocking antibodies, this presents some limitations including high costs, low tissue penetration, unclear mode of action *in vivo*, cytotoxicity, and affinity for inhibitory Fc receptors which reduces their overall efficacy (87). Soluble decoys that can interfere with specific ligand-receptor interactions and are small enough to achieve good biodistribution in solid tumors present more attractive options. Extracellular domains of Notch1 that uniquely interact with Delta-like or Jagged classes of ligands have been used to develop Notch decoys achieving ligand-specific inhibition (28, 86, 88). A fragment of Notch1 ECD comprised of EGF repeats 10 to 24 (N1_10–24_) could selectively inhibit Notch1-JAG interactions without interfering with Notch1-DLL interactions indicating competitive binding to Jagged ligands. N1_10–24_ demonstrated potent antitumor effects in various murine tumor models by reducing angiogenic sprouting and disruption of tumor endothelium, both of which are phenotypes associated with JAG1-driven Notch signaling. Similarly, the Notch1 ECD fragment comprised of EGF repeats 1 to 13 (N1_1–13_) could specifically inhibit DLL4-mediated Notch signaling effects leading to hyper-sprouting and poor perfusion. On the other hand, a larger Notch1 decoy, N1_1–24_, recapitulated the effects of inhibiting either JAG1 or DLL4 or both, depending on the tumor microenvironment and *in vitro* angiogenesis model used.

Soluble inhibitory receptor-derived decoys have been reported for Notch3 as well. Distinct, short peptides derived from EGF repeats 7–10 and 21–22 of Notch3 bound directly to JAG1 (89). The ligand-binding domains of Notch3 were distinct from those of Notch1 despite the high sequence similarity in conserved EGF repeats. The peptide forms as well as recombinant immunoglobulin Fc chimeras (IgG-Fc) of Notch3-derived peptides were able to induce apoptosis in tumor cells, preferentially reduced Notch3 activation and the expression of Notch3-specific target *Hey1*. Peptide-IgGFc chimeras could also suppress tumor growth in a Notch3-driven human lung cancer xenograft model.

Thus, certain regions of Notch receptor extracellular domains that uniquely interact with different ligand classes and paralogs can be used to design soluble inhibitory decoys with high specificity.

Full-length or partial extracellular domains of Notch ligands could be also be used to modulate ligand-specific Notch signaling events. Soluble monomeric fragments comprised of the DSL and first two EGF repeats of DLL1 (sDLL1) and the first five N-terminal domains of JAG1 (sJAG1_N–E3_) could selectively inhibit Notch1-DLL1 and Notch1-JAG1 interactions, respectively (6). The sDLL1 fragment attenuated *in vitro* T cell proliferation in cocultures with DLL1-bearing dendritic cells, indicating its potential ability to impair T cell responses by blocking endogenous DLL1. A short synthetic peptide derived from DSL region of JAG1 spanning residues 188–204 demonstrated Notch activation driving keratinocyte differentiation *in vitro* in its soluble form (90). Portions of the DSL domain of human JAG1 as well as the complete extracellular region of human JAG1 ligands could function as activators affecting differentiation of myeloid progenitors (91). In contrast, soluble purified human Jagged1-immunoglobulin IgG1 Fc chimera protein inhibited growth of myeloid colonies and macrophage progenitors from human cord blood, indicating its inhibitory properties (92). Therefore, the exact sequence of Notch ligand extracellular domains used (partial fragment versus full length ECD, specific portions of extracellular domains) could determine whether the soluble ligand forms act as activators or inhibitors. This might be driven by the area and structural conformation of ligand-receptor interface being bound by the soluble ligands. Detailed structural and binding studies need to be done to evaluate the mechanistic aspects of soluble ligands as Notch modulators.

Ligand multivalency is commonly mimicked by immobilizing the ligands on culture plates prior to seeding cells or by pre-clustering of ligand immunoglobulin Fc fragment chimeras by anti-Fc antibodies (2, 93). While plate-bound ligands can provide multimeric ligand stimulus, their use is restricted to *in vitro* applications. For *in vivo* administration, pre-clustered ligands provide a more suitable format. Further complexing anti-Fc antibodies is possible by tagging them with biotin, FLAG or other non-immunogenic short peptides or affinity tags (94) and using anti-tag antibodies to in turn complex those. This would greatly increase the valency of ligand being provided and could be used for pharmacological stimulation of ligand-specific responses. A multiplexed reagent called clustered DLL1 (cDLL1) was developed which is comprised of three components: a chimera of full length murine or human DLL1 and Fc region of IgG_2A_, biotinylated anti-IgG_2_Fc antibody, and NeutrAvidin (a deglycosylated version of avidin with unaltered affinity to biotin) (1). This produces a tertiary complex with multiple ligand extracellular domains being available for Notch activation. cDLL1 administration to tumor-bearing mice improved antigen-specific CD8^+^ T cell responses and attenuated tumor growth in preclinical murine models of lung cancer. Multivalent DLL1 stimulus provided by cDLL1 could enhance CD8^+^ T effector-memory cells and reduce the number of regulatory T cells in spleen.

Apart from providing *in vitro* therapeutic agents, plate-bound, cell-expressing, and multimerized Notch ligands could thus be used in various multivalent formats for cancer treatment.

### Affinity-Modifying Compounds

Carbohydrate moieties at the ligand-receptor interface in *trans* interactions can influence canonical ligand-mediated Notch receptor activation via steric effects (95–97). L-fucose analogs that could be directly incorporated into Notch EGF repeats can be exploited to manipulate Notch receptor binding to cognate ligands. Peracetylated forms of *O*-Fucose, 6-alkynyl and 6-alkenyl fucose, act as substrates by Pofut-1 and can differentially modulate ligand binding (98). Fucose analogs incorporated into Notch1 EGF repeats inhibit trans interactions with DLL1 and DLL4 whereas interactions with JAG1 remain unaffected. Mutational and structural analysis revealed that fucosylation at Notch EGF8 is the site contributing to steric clashes and subsequent ablation of interactions with Delta-like ligands. This can be explained by a higher sensitivity of Delta-like ligands to Notch post-translational modifications compared to the Jagged class of ligands.

Notch ligand-based and affinity-modifying reagents thus offer the benefit of specific targeting along with fine-tuning and versatility to activate or inhibit Notch activity in a ligand-specific manner.

The following sections summarize evidence from preclinical and clinical studies that provided substantial evidence in favor of Notch ligand-based moieties as immunomodulatory agents driving anti-tumor T cell functions.

## Restoring DLL1-Specific Notch Signaling Can Reverse Impaired T Cell Development in Tumor-Bearing Mice

Tumor presence alters a number of cytokine-mediated intracellular signaling pathways, expression of chemotactic ligands and receptors by thymic populations (99–101). This results increased apoptosis of TECs, dysregulated lineage-commitment checkpoints, diminished TCR repertoire and low thymic output, all of which ultimately dampen immunosurveillance and promote tumor escape. In immature DN2 T cell subsets, CCR7 is a target of Notch1 and is important for the migration of developing T cell precursors through the thymic cortex to medulla (102–104). Reduction in expression levels of Notch1 and its targets in thymic pre-T cells of tumor-bearing mice is mediated by IL-10 produced by thymic epithelial cells (TECs) (45). This is associated with an upregulation in of Ikaros and IRF8 signaling which shunts the developing pre-T cell toward differentiating into dendritic cells. A network of interactions between Notch, Wnt, Ikaros, and IL10 (among several others) is involved in determining the balance between T and myeloid lineage commitment under normal physiologic conditions (105–108). Given the indispensable role of Notch in ensuring normal thymic T cell development, therapeutic interventions to restore Notch activity in thymic and peripheral T cells can promote antitumor immunity (109, 110).

Advanced stage cancer patients have high mean serum levels of vascular endothelial growth factor (VEGF) compared to healthy humans. Mice infused with VEGF to mimic this pathophysiology showed thymic atrophy and decreased percentage of peripheral T cells in their spleen and lymph nodes (46, 111). This effect was coupled to a significant decrease in the number of CD4^+^CD8^+^ thymic populations. The reduction in CD4^+^CD8^+^ numbers was not due to an induction of thymocyte apoptosis or inhibition of thymocyte development, as the VEGF-exposed thymic cells could develop normally in mice without tumors and in *in vitro* fetal thymic organ cultures. Administration of anti-VEGFR2 but not anti-VEGFR1 antibody restored normal hematopoiesis revealing a mechanistic link between tumor derived VEGF and impaired peripheral immunity (112). The thymic atrophy observed in tumor-bearing mice could be a consequence of a pre-thymic event such as a VEGF-mediated block in emigration of thymic progenitors from the bone marrow.

Compared to age-matched controls, tumor-bearing mice have low DLL1 and DLL4 expression levels in bone marrow cells as well as low splenic T:B cell ratios. When VEGF-infused mice were irradiated and received bone marrow progenitors overexpressing DLL1, the inhibitory effects on T cell development were reversed, indicating that DLL1 stimulus alone is sufficient to resuscitate VEGF-driven impaired antitumor immunity. In order to mimic the effects of BM transplantation with DLL1-overexpressing hematopoietic precursors, the more pharmacologically relevant multivalent DLL1 form (cDLL1 – described in the previous section) was employed (5). Administration of cDLL1 significantly lowered tumor burden in treated mice compared to untreated tumor-bearing controls. Tumor regression was T-cell mediated, as was seen with the loss of cDLL1 efficacy in tumor-bearing *Rag1*^–/–^ mice and mice receiving anti-CD8 antibody to deplete CD8^+^ T cells. This was associated with increased number of antigen-specific memory T cells, improved IFNγ production, and higher intracellular pSTAT1&2 in differentiated T cells. Additionally, the transcript levels of T-bet were significantly higher in CD4^+^ T cells after cDLL1 administration, providing direct evidence of tumor attenuating Th1 responses being enhanced (112–115). Stimulation of Notch signaling in effector CD8^+^ T cells was also able to achieve tumor regression in mutant EGFR^L858R^ oncogene-driven tumor models. Patients with EGFR-driven non-small cell lung cancers treated with tyrosine kinase inhibitors (TKI) such as erlotinib eventually acquire drug resistance (116–118). The TKI treatment also shapes the tumor microenvironment leading to an upregulation of PD-L1 expression (119–121). Given the low response rate of EGFR-mutant tumors to ICI treatment (122–124), DLL1-mediated enhancement of Type I immune responses might provide therapeutic benefit when used in combination with ICI.

Different Notch ligand-receptor interactions can result in distinct downstream outcomes hence while DLL4 stimulation promotes angiogenesis, DLL1 signaling does not (125–127). Concurrently, the administration of cDLL1 to tumor-bearing mice did not result in vascular defects and, in fact, significantly decreased tumor vascularization.

In this manner, Notch ligand-based therapeutics can selectively stimulate helper and effector immune functions with high ligand and contextual specificity. Multivalent DLL1-derived Notch activators could thus potentially provide clinically relevant immunotherapeutic agents to overcome thymic atrophy and impaired T cell functions.

## Regulatory T Cell Functions Can Be Modulated in a JAG1-Specific Manner

Interaction of T cells with APCs is necessary to induce effector T cell function and differentiation by providing TCR stimulus from cognate peptide-bound MHC (signal 1) and costimulatory CD28 (signal 2) (128–130). Several findings have revealed the additional role of interactions between specific Notch ligands presented by DCs and Notch receptors on T-cells in providing critical activation, differentiation, and polarization signals.

Adoptive transfer of antigen-pulsed, Jagged1 (JAG1)-expressing DCs inhibited established immune responses in immunized mice. This inhibition was CD4^+^ T cell-specific and long lived (131). With JAG1-expressing DC administration, peripheral naive CD4^+^ T cells were found to differentiate into regulatory cells. These cells could induce antigen-specific tolerance when transferred into naïve hosts. Similar effects were seen in human peripheral naïve blood cells; stimulation of CD45RA^+^ naïve T cells by allogeneic antigen-presenting cells overexpressing JAG1 resulted in reduced production of IFNγ, IL-2 and IL-5. The activated cells upregulated TGFβ and inhibited proliferative and cytotoxic immune responses in freshly stimulated lymphocyte cultures (132). This reveals the molecular basis of regulatory T cell induction when naïve cells are activated by JAG1-borne APCs. Further investigation revealed that the immunosuppression was antigen-specific and affected both CD4^+^ and CD8^+^ T cells (133).

Mouse bone marrow cells could be differentiated into tolerogenic dendritic cells by culturing them in the presence of GM-CSF (GM-BMDCs). GM-BMDCs were found to express JAG1, essential for induction of regulatory T cell phenotype in CD4^+^ T cells (134). Abrogating JAG1-Notch interactions by using anti-JAG1 blocking antibodies suppressed T_reg_ proliferation. Similar results were obtained by shRNA-mediated knockdown of JAG1 in murine bone marrow mesenchymal stromal cells (MSC) (135). CD4^+^ T cells cocultured with JAG1-expressing MSC differentiated into tolerogenic T_reg_ cells capable of producing anti-inflammatory cytokine, IL-10. T_regs_ thus obtained could also protect against inflammation *in vivo* in a mouse model of allergen-induced airway pathology.

Since DCs expressing JAG1 exhibit a tolerogenic potential, they could be used in a transplantation setting to inhibit immune responses and prolong allograft survival. Indeed, when JAG1-overexpressing DCs were used in along with CD40 blocking antibody, murine allograft heart transplants were better tolerated in recipient mice (136). This was achieved by induction of alloantigen-specific T cell suppression and upregulation of TGFβ and FoxP3-expressing T_reg_ numbers driven by JAG1-Notch interactions expressed by transferred DCs and host T cells, respectively. While JAG1-driven Notch activation of host T cells could attenuate Th1 responses, it did not effect Th2 differentiation. By employing JAG1-transduced DCs, this study could provide mechanistic insights into the specific source and functions of Notch ligands. It is worth noting that overexpression of JAG1 could lead to ligand being in far in excess of receptors available on T cells and can diminish Notch activation owing to reduced ligand trans-endocytosis (137, 138).

Further evidence that T_reg_ -mediated suppression of effector T cell responses was mediated by Notch came from a systematic lineage-specific deletion of Notch pathway components in T_regs_. Targeted deletion of *Pofut1*, *Rbpj* and *Notch1* enhanced T_reg_ cell frequency and decreased CD4^+^ and CD8^+^ T-cell immune responses (139). On the other hand, overexpression of a constitutionally active Notch1 intracellular domain in T_reg_ cells resulted in autoimmunity, skewing to a Th1 phenotype and apoptosis of regulatory T cells. Notch inhibition appears to dictate the balance between inflammatory effector T cells and tolerant regulatory T cells.

Tissue tissue-specific genetic ablation of Jagged1 and systemic administration of soluble inhibitory JAG1 provided further proof of ligand-mediated Notch activation in T_regs_ (6). CD11c-specific ablation of JAG2 did not have any effects on IFN-γ production but significantly decreased IL-4 production by activated T cells. On the other hand, CD11c-specific deletion of DLL1 resulted in accelerated tumor growth in murine tumor models coupled to a reduction in CD8^+^ T cell activation and reduced differentiation of antigen-specific cytotoxic T cells and memory cells. Tumor-bearing mice treated with inhibitory monomeric soluble JAG1 (sJAG1) showed a significant reduction in tumor burden concurrent with a decrease in splenic T_reg_ cell numbers. This was associated with low tumor infiltration of CD11c^+^Gr1^+^ cells, thereby providing further evidence of JAG1 as a factor mediating immunosuppressive tolerogenic responses. *In vitro* T:DC coculture experiments in the presence of sJAG1 could also downregulate the expression of PD-1 on CD8^+^ T effector memory cells.

Taken together, these studies provide a strong evidence in favor of specifically targeting JAG1 to modulate regulatory T cell functions. The use of well designed, soluble inhibitory JAG1 decoys could provide a therapeutic edge in the context of enhancing antitumor immune responses and attenuating immunosuppression. Distinct ligand-specific effects provide a great opportunity to avoid undesirable effects associated with pan-Notch inhibition.

## Concluding Remarks and Future Perspectives

From the perspective of both basic and applied immunology, study of Notch signaling in immune subsets can provide valuable insights into the management and cure of metastatic solid tumors that are recalcitrant to conventional treatments. As the field of immunology progresses, so will our understanding of the role that Notch plays in immune cell function and regulation. This can be accomplished by interdisciplinary and complementary techniques such as tissue and lineage-specific genetic ablation, biochemical and molecular modulation of ligand-receptor interactions, evaluation of antigen specific immune responses and computational analysis of large patient datasets. More fundamental approaches such as investigating ligand/receptor redundancies, effector differentiation by cytokines in combination with ligand-specific Notch activation and non-canonical Notch signaling are also needed. Outcomes from current therapeutic regimens can be improved by using Notch-ligand based reagents in combination with or prior to checkpoint blockade to prime the immune system. Preclinical studies using Notch ligand-derived selective activators and inhibitors also provide mechanistic insights into how the immune system can be modulated in a ligand-specific manner in cancer and other immunopathological conditions. Such agents constitute a novel class of immunomodulatory drugs addressing unmet medical needs.

## Author Contributions

All authors listed have made a substantial, direct and intellectual contribution to the work, and approved it for publication.

## Conflict of Interest

The authors declare that the research was conducted in the absence of any commercial or financial relationships that could be construed as a potential conflict of interest.

## Abbreviations

CSL: CBF-1, suppressor of hairless, lag-2
DLL: Delta-like ligand
ECD: extracellular domain
GSI: γ -secretase inhibitor
HSC: hematopoietic stem cell
ICI: immune checkpoint inhibitor
JAG: Jagged ligand
LLC: Lewis lung carcinoma
MAML1: mastermind-like protein 1
NECD: Notch extracellular domain
NICD: Notch intracellular domain
NRR: negative regulatory region
NSCLC: non-small cell lung cancer
PEST: peptide sequence rich in proline (P), glutamic acid (E), serine (S) and threonine (T)
RBP-Jκ: recombination signal binding protein for immunoglobulin Kappa J region
SAHM1: Stapled α -helical peptides derived from mastermind-like protein 1
T-ALL: T-cell acute lymphoblastic leukemia
TME: tumor microenvironment.
